# Retraction: Dominant Negative Mutants of *Bacillus thuringiensis* Cry1Ab Toxin Function as Anti-Toxins: Demonstration of the Role of Oligomerization in Toxicity

**DOI:** 10.1371/journal.pone.0345629

**Published:** 2026-03-25

**Authors:** The PLOS One Editors

After this article [1,2] was published, concerns were raised regarding Figs 1, 2, and 4. Specifically,

- In Fig 1, when levels are adjusted to visualize the background, there appears to be a vertical discontinuity between the T143D+ and D136N/T143D- lanes.
- In all panels of Figs 2B and 2C in [1] and [2] there appear to be multiple horizontal and vertical discontinuities, areas that appear to be discontinuous with their adjacent background regions, and multiple repetitive areas within each panel.
- In Fig 4A, there appears to be an irregularity above the current-time output in the E129K/D136N Membrane pellet panel.

In light of the above concerns that question the reliability and integrity of the reported results and conclusions, the *PLOS One* Editors retract this article [1,2].

CRA, CMG, SP, MS, and AB did not agree with the retraction and stand by the article’s findings. LEZ, NJJ, and LM either did not respond directly or could not be reached.
